# Heritable and inducible gene knockdown in astrocytes or neurons *in vivo* by a combined lentiviral and RNAi approach

**DOI:** 10.3389/fncel.2014.00062

**Published:** 2014-03-19

**Authors:** Fabrice Heitz, Torbjörn Johansson, Karsten Baumgärtel, Rreze Gecaj, Pawel Pelczar, Isabelle M. Mansuy

**Affiliations:** ^1^Brain Research Institute, Medical Faculty of the University of Zürich and Department of Biology of the Swiss Federal Institute of TechnologyZürich, Switzerland; ^2^Institute of Pharmacology and Toxicology, Medical Faculty of the University of ZürichZürich, Switzerland; ^3^Dorris Neuroscience Center, The Scripps Research InstituteLa Jolla, CA, USA; ^4^Institute of Laboratory Animal Science, University of ZürichZürich, Switzerland

**Keywords:** *in vivo* gene knockdown, conditional gene knockdown, shRNA, Cre-lox genetics, lentivirus-mediated transgenesis

## Abstract

Gene knockout by homologous recombination is a popular method to study gene functions in the mouse *in vivo*. However, its lack of temporal control has limited the interpretation of knockout studies because the complete elimination of a gene product often alters developmental processes, and can induce severe malformations or lethality. Conditional gene knockdown has emerged as a compelling alternative to gene knockout, an approach well-established *in vitro* but that remains challenging *in vivo*, especially in the adult brain. Here, we report a method for conditional and cell-specific gene knockdown in the mouse brain *in vivo* that combines Cre-mediated RNA interference (RNAi) with classical and lentivirus-mediated transgenesis. The method is based on the inducible expression of a silencing short hairpin RNA (shRNA) introduced in mice by lentivirus-mediated transgenesis, and on its activation by excision of a floxed stop EGFP reporter with an inducible Cre recombinase expressed in astrocytes or in neurons. This dual system should be of broad utility for comparative studies of gene functions in these two cell types *in vivo*.

## Introduction

Genetic models in the mouse have significantly advanced the understanding of gene functions, both in physiological and pathological conditions. Most of the existing models have been generated by genetic manipulations that lead to a gain- or loss-of-function of the candidate gene (Aronoff and Petersen, 2006). This is usually achieved with vector-based overexpression systems, or by gene targeting like gene knockout or knockin (Capecchi, 2005; Glaser et al., 2005). Recently, RNA interference (RNAi) has emerged as an alternative approach with the advantage of decreasing rather than fully eliminating the expression of target gene(s) (McManus and Sharp, 2002; Paddison and Hannon, 2002). RNAi-based gene knockdown relies on short double-stranded RNAs (19–21 nucleotides) that target complementary mRNA sequences, and promote their degradation or inhibit their translation (McManus and Sharp, 2002; Dykxhoorn et al., 2003). It can be used in rodents by local or systemic delivery of short-interfering RNAs (siRNAs) (Thakker et al., 2004), or through virus-based shRNA expression vectors (Ralph et al., 2005; Singer et al., 2005; Sapru et al., 2006). Although this approach can be extremely useful, its efficiency is subjected to variability and depends on several factors, in particular the degree of sequence matching with the target DNA, and the level of expression of the shRNA and the targeted gene. Further, even if it has been used successfully in cell culture (Elbashir et al., 2001; Brummelkamp et al., 2002; Lee et al., 2002), it remains challenging *in vivo.* Thus, overall, genetic systems for gene silencing have been reported in the literature (Rubinson et al., 2003; Tiscornia et al., 2003; Coumoul et al., 2005; Szulc et al., 2006; Dickins et al., 2007; Delic et al., 2008; Seidler et al., 2008) but are often difficult to use due to their limitations such as insufficient temporal and spatial control, and unspecific off-target effects (Scacheri et al., 2004).

Here, we describe a combinatorial genetic approach to achieve stable, heritable, and inducible gene knockdown in astrocytes or neurons in the adult mouse brain *in vivo*. The approach is based on the combination of the Cre-loxP system (Sauer and Henderson, 1989) and a lentiviral vector (Ventura et al., 2004) that allow the conditional expression of a short hairpin RNA (shRNA) in specific cells in the brain and its tracing by an EGFP reporter. We provide a proof of principle for the efficiency and power of the method for *in vivo* and *ex vivo* applications.

## Results

### Establishment and validation of shRNA-mediated gene knockdown *in vitro*

**Figure 1:**
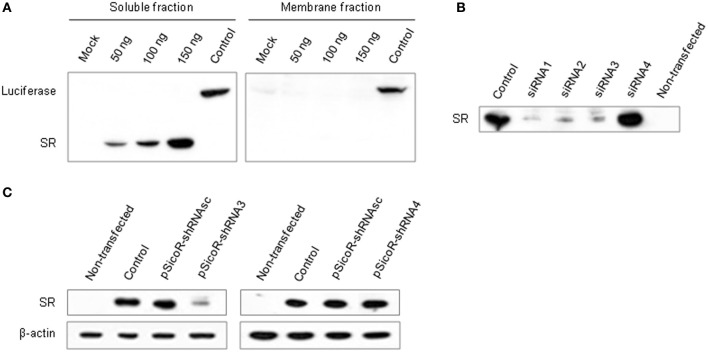
**Establishment of siRNA-dependent knockdown of a target gene (SR) *in vitro*. (A)** Western blot showing luciferase and SR expression in Hek293T cells transfected with 50, 100, and 150 ng of pCMV-Tag2-SR encoding SR fused to an epitope peptide (flag). Mock, empty pCMV-Tag2 plasmid; Control, pCMV-Tag2 plasmid carrying a tagged firefly luciferase. Optimal cell delivery was determined by comparing siRNA-Alexa 488 fluorescence distribution (not shown). **(B)** Silencing efficiency of SR siRNAs. Western blot showing SR expression in Hek293T cells co-transfected with pCMV-Tag2-SR and siRNAs 1–4, or control pCMV-Tag2 (Control). Non-transfected cells were used as negative control. **(C)** SR knockdown by shRNA3. Western blot showing SR expression in Hek293T cells co-transfected with pCMV-Tag2-SR, pSicoR-shRNAsc encoding a scrambled shRNA and pSicoR-shRNA3 (left panel), or pSicoR-shRNA4 (right panel). pCMV-Tag2-SR was co-transfected with the empty vector pCDNA3.1 as control. β-actin was used as loading control.

To establish a method for gene knockdown in astrocytes or neuronal cells in the mouse brain, we designed interfering small RNAs against a candidate gene known to be expressed in both cell types. We selected the gene coding for serine racemase (SR), a cytoplasmic enzyme that synthesizes D-serine from L-serine (Wolosker, 2007; Baumgart and Rodriguez-Crespo, 2008). siRNA duplexes matching the SR sequence were designed and their efficiency to knockdown SR expression was evaluated *in vitro*. A vector for expressing a flag-tagged SR (pCMV-Tag2-SR) was cloned, and transiently transfected into human embryonic kidney cells (Hek293T). In the resulting transfected cells, SR protein was expressed, and was enriched in the soluble fraction. The expression level was proportionate to the concentration of the vector (Figure 1A). Cells expressing SR were then transfected with either one of four siRNAs (siRNA1–4) against SR (Table S1A). Three siRNAs (1–3) significantly reduced SR expression in the transfected cells and led to a consistent SR knockdown (Figure 1B). siRNA3 was selected for use *in vivo* (Figure S1A). SiRNA4 did not have any effect on SR expression.

Stable RNAi is best obtained *in vivo* using vectors expressing shRNAs (double-stranded) that are processed into siRNAs by the cellular machinery (McManus and Sharp, 2002; Dykxhoorn et al., 2003). To constitutively express an shRNA matching siRNA3 *in vivo,* we used a pSicoR vector (Ventura et al., 2004) and first tested its efficiency in cell culture. Consistent with the results obtained with siRNA duplexes, shRNA3 induced a significant knockdown of SR in three independent replicates (Figure 1C and Table S1B). It resulted in a mean decrease in SR expression of 48% (±20%) and 39% (±23%) with 1:1 and 1:3 plasmid ratios, respectively (pSicoR-shRNA3:pCMV-Tag2-SR) (Figure S1B). The effect was specific to SR since no change in β-actin expression was detected. Control scrambled shRNA or shRNA4 matching siRNA4 had no effect on SR expression.

### Generation of mice carrying a conditional shRNA3 expression vector by lentivirus-mediated transgenesis

**Figure 2:**
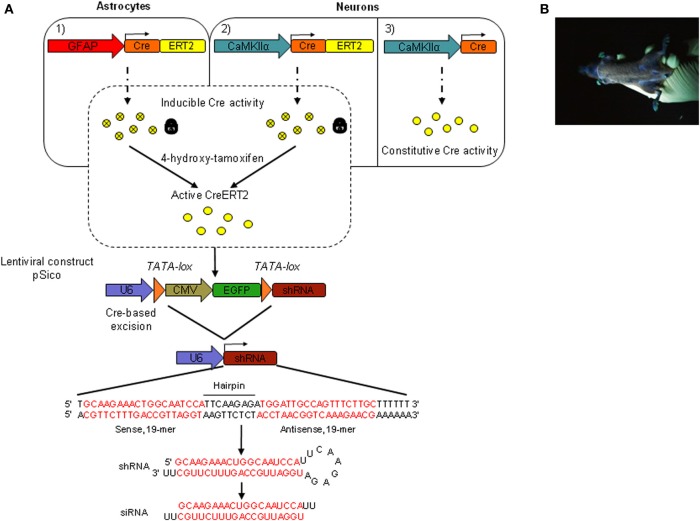
**Inducible and cell-specific gene knockdown *in vivo*. (A)** Schematic representation of the genetic system used to achieve inducible and astrocyte- or neuron-specific shRNA expression in the adult mouse brain. pSico-shRNA3 × GFAP-CreERT2 (1) and pSico-shRNA3 × CaMKIIα-CreERT2 mice (2) express a Cre recombinase fused to a mutated version of the human estrogen receptor ligand-binding domain (ERT2). CreERT2 requires 4-hydroxytamoxifen (active tamoxifen metabolite) to be active. pSico-shRNA3 × CaMKIIα-Cre mice (3) express a constitutively active Cre recombinase. CreERT2/Cre expression is under the control of either the astrocyte-specific GFAP promoter (1) or the forebrain neuron-specific CaMKIIα promoter (2). Cre or tamoxifen-activated CreERT2, excise a stop/reporter cassette flanked by loxP sites from the pSico lentivirus-based vector and switch on shRNA expression. The synthesized shRNA is then processed into a siRNA by the cellular machinery, with a predicted siRNA sequence complementary to the targeted SR mRNA (in red). U6, Pol III promoter; CMV, pol II promoter; EGFP, enhanced green fluorescent protein; shRNA, small hairpin RNA; siRNA, small interfering RNA. **(B)** Picture of a transgenic founder carrying the pSico-shRNA3 lentiviral construct and showing ubiquitous EGFP expression, as visualized under UV light.

To achieve conditional expression of shRNA3 in the mouse brain *in vivo*, we generated transgenic mice by lentivirus-mediated transgenesis. This method was reported to be highly efficient, and to allow the rapid and simultaneous generation of multiple independent transgenic lines (Lois et al., 2002). To express shRNA3, we used a pSico lentiviral vector that was previously used for conditional gene knockdown based on the Cre-loxP system (Sauer and Henderson, 1989; Ventura et al., 2004). An oligonucleotide encoding shRNA3 was inserted downstream of the ubiquitous U6 RNA polymerase III promoter and of a floxed stop/reporter cassette encoding EGFP under the ubiquitous CMV promoter (Figure 2A). Lentivirus carrying the pSico-shRNA3 construct was produced in Hek293T cells and used to generate transgenic mice by injection of the lentiviral particles into the perivitelline space of fertilized mouse oocytes. Lentiviral transgenesis was achieved using transgenic oocytes carrying a CreERT2 (an inducible form of Cre) transgene driven by a glial fibrillary acidic protein (GFAP) promoter. These mice which carry GFAP-CreERT2 express CreERT2 in astrocytes and were generated by classical pronuclear injection (Feil et al., 1996; Hirrlinger et al., 2006). The goal of this strategy was to avoid the need for subsequent back-crossing of individual pSico-shRNA3 founder mice with mice carrying the GFAP-CreERT2 transgene. In the resulting double transgenic animals, CreERT2 is expected to lead to the excision of the floxed/stop EGFP reporter cassette from pSico-shRNA3 in the presence of tamoxifen, and to shRNA3 expression only in astrocytes.

Eleven pups (F0) born from the lentivirus-injected oocytes after transplantation in foster mothers were genotyped, and eight were found to carry the pSico-shRNA3 vector (transgenesis efficiency of 72.7%). This high ratio of transgenesis is consistent with the expected high rate of provirus integration as reported earlier (Lois et al., 2002). The visualization of EGFP signal revealed that four of the eight founder mice had strong and widespread EGFP expression (Figure 2B). The eight founders were then used to establish transgenic lines by breeding with wild-type C57BL/6 mice. On average, 63.5% of the first generation offspring (F1) was positive for pSico-shRNA3, consistent with the expected integration of the proviral transgene at multiple chromosomal loci, including in germ cells (Lois et al., 2002). Further, on average, half of the progeny was positive for GFAP-CreERT2 (normal Mendelian inheritance for a transgene integrated in a single locus) (Table S2A). Two lines (1 and 3) were further bred to wild-type C57BL/6 mice down to the third generation (F3), and one line (line 2) down to the fifth generation (F5). These lines showed a transmission ratio of the pSico-shRNA3 transgene close to 50% by the third generation, suggesting segregation of the transgene to a single locus after two backcrosses. It is noteworthy that the reduction of integration sites down-to-one occurred over only two generations, possibly because the virus was injected at a low titer and integrated only into few loci.

**Figure 3:**
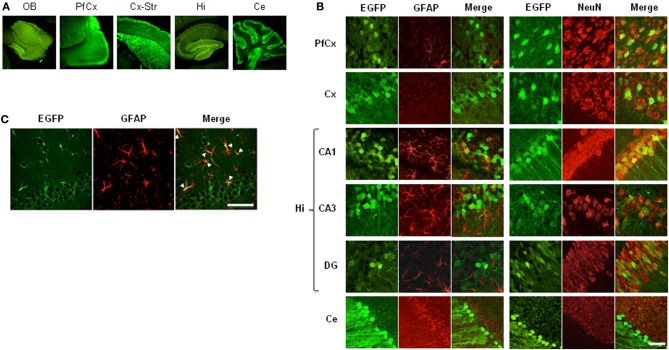
**EGFP expression in mice carrying pSico-shRNA3. (A)** Immunostaining showing EGFP expression (green) in olfactory bulb (OB), prefrontal cortex (PfCx), cortex and striatum (Cx-Str), hippocampus (Hi), and cerebellum (Ce). **(B)** EGFP co-immunostaining with the astroglial marker GFAP (red, left panels) or the neuronal marker NeuN (red, right panels) in PfCx, Cx, Hi [area CA1, CA3, dentate gyrus (DG)], and Ce. Scale bar: 10 and 40 μm for cerebellum. **(C)** EGFP expression in astrocytes in hippocampal area CA1 by co-localization with GFAP after antigen unmasking. Scale bar: 10 μm.

Once adult mice carrying the lentiviral transgene were obtained, we determined the region- and cell-specificity of EGFP expression in pSico-shRNA3 mice by immunostaining. EGFP expression was detected throughout the brain (Figure 3A); it was weak in astrocytes but strong in many neurons including pyramidal cells in the cortex (layer II–III, neuronal cell bodies, and axonal projections) and hippocampus area CA1-CA3, granule cells in the dentate gyrus, the cerebellum and the olfactory bulbs, spiny neurons in the striatum, and Purkinje neurons (Figures 3B, S2A). Since EGFP signal was weak in astrocytes, we carried out further immunostaining using an antigen unmasking method to confirm the signal. This staining showed a much stronger and consistent EGFP expression in astrocytes in multiple brain regions including prefrontal areas, cortex, hippocampus, dentate gyrus (Figure 3C), confirming that EGFP is expressed in both glial and neuronal cells.

### Stable, inducible, and cell-specific knockdown in the adult mouse brain *in vivo*

Next, we evaluated the efficiency and specificity of SR knockdown by shRNA3 *in vivo*. To test the knockdown in astrocytes or neurons in the adult brain, we generated double transgenic mice carrying pSico-shRNA3 and either, GFAP-CreERT2 for knockdown in astrocytes, or CaMKIIα promoter-CreERT2 or CaMKIIα promoter-Cre for knockdown in forebrain neurons.

#### Inducible astrocyte-specific SR silencing in the adult mouse brain

**Figure 4:**
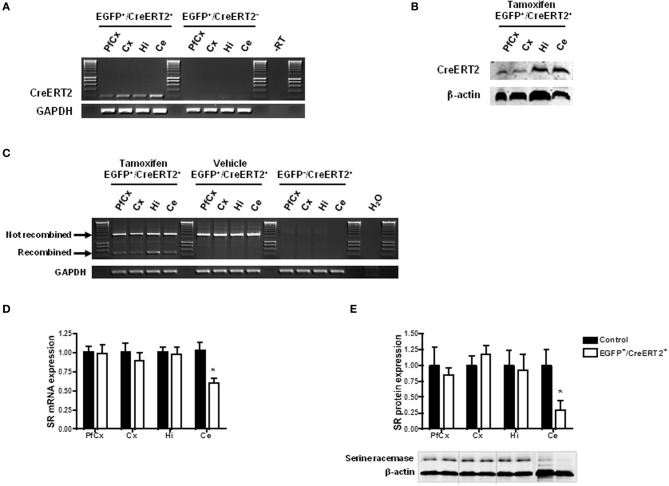
**Inducible and astrocyte-specific gene knockdown in the adult mouse brain. (A)** RT-PCR showing CreERT2 expression in pSico-shRNA3 × GFAP-CreERT2 (EGFP^+^/CreERT2^+^) mice. No expression was detected in the absence of CreERT2 (EGFP^+^/CreERT2^−^ control mice). –RT, Non-reverse transcribed sample. **(B)** Immunoblot analysis of CreERT2 protein in pSico-shRNA3 × GFAP-CreERT2 (EGFP^+^/CreERT2^+^) mice. **(C)** PCR showing Cre-mediated recombination induced by tamoxifen treatment in different brain structures in pSico-shRNA3 × GFAP-CreERT2 mice. No recombination was detected in mice not treated with tamoxifen (vehicle). EGFP^−^/CreERT2^+^ mice were used as negative control, and GAPDH as loading control. **(D)** Real-time quantitative RT-PCR showing a significant decrease in SR mRNA expression in cerebellum in pSico-shRNA3 × GFAP-CreERT2 adult mice (EGFP^+^/CreERT2^+^, *n* = 8) compared to control mice (EGFP^+^/CreERT2^−^ and EGFP^−^/CreERT2^+^, *n* = 8). β-actin was used as internal control. **(E)** SR protein level is significantly decreased in cerebellum of pSico-shRNA3 × GFAP-CreERT2 adult mice (EGFP^+^/CreERT2^+^, *n* = 5) relative to control mice (EGFP^+^/CreERT2^−^ and EGFP^−^/CreERT2^+^, *n* = 5). Immunoblot analysis with 15 μg of total proteins loaded for prefrontal cortex (PfCx), cortex (Cx), hippocampus (Hi), and 25 μg for cerebellum (Ce). β-actin was used as a loading control. ^*^p < 0.05.

**Figure 5:**
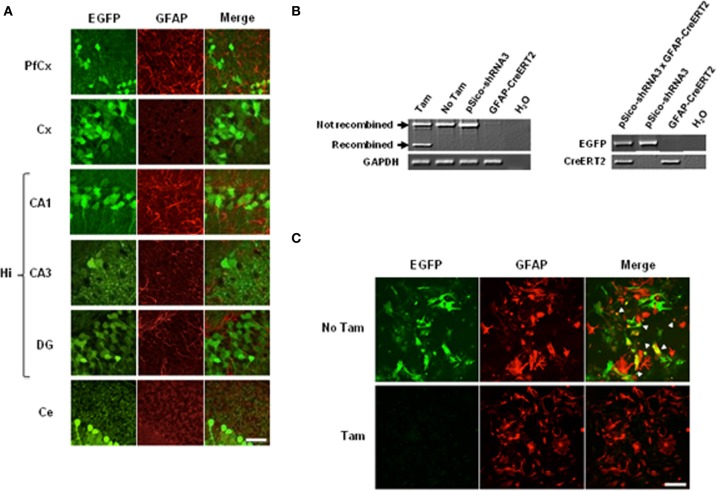
**Cre-mediated recombination in astrocytes and neurons. (A)** Immunohistochemical analysis of EGFP expression in tamoxifen- treated pSico-shRNA3 × GFAP-CreERT2 mice. EGFP (green) and GFAP (red) co-immunostaining in prefrontal cortex (PfCx), cortex (Cx), hippocampus (CA1, CA3, DG), and cerebellum (Ce). Scale bar: 40 μm. **(B)** PCR showing Cre-mediated recombination (left panel) in pSico-shRNA3 × GFAP-CreERT2 primary astrocytes by 4-hydroxytamoxifen treatment (Tam). No recombination is detected in non-treated cells (No Tam) or in astrocytes from mice carrying pSico-shRNA3 and GFAP-CreERT2 alone. Genotyping PCR from pSico-shRNA3 × GFAP-CreERT2, pSico-shRNA3, and GFAP-CreERT2 astrocytes (right panel). **(C)** EGFP and GFAP co-staining in pSico-shRNA3 × GFAP-CreERT2 astrocytes treated with 4-hydroxytamoxifen (Tam) compared to non-treated astrocytes (No Tam).

In order to confirm that the conditions were gathered to achieve SR knockdown in astrocytes, we first examined the profile and level of expression of CreERT2 in the mice carrying pSico-shRNA3 and GFAP-CreERT2. Quantitative real-time PCR revealed that CreERT2 mRNA was expressed throughout the brain, including in prefrontal and cerebral cortex, hippocampus, and cerebellum (Figure 4A). The observed pattern of expression was consistent with that described previously in GFAP-CreERT2 mice (Hirrlinger et al., 2006). But despite broad mRNA expression, CreERT2 protein was detected only in the hippocampus and cerebellum (with very low level in prefrontal and cerebral cortex) (Figure 4B), possibly due to posttranscriptional regulation. Consistently, recombination after tamoxifen treatment was detected in the hippocampus and cerebellum but only weakly in cortical areas (Figure 4C). In the cerebellum, CreERT2-dependent recombination resulted in a decrease in SR mRNA (39.49% ± 3.83) and protein (69.82% ± 34.05), indicating functional shRNA3 expression and gene silencing (Figures 4D,E). However, in the hippocampus, no SR knockdown was detected despite CreERT2 expression. Although surprising, this result is consistent with a previous report showing a dissociation between gene recombination and silencing (Turlo et al., 2010). Importantly, the observed recombination was specific to astrocytes and did not occur in neurons as EGFP expression was unaltered in neurons after tamoxifen treatment (Figure 5A). Finally, no recombination was detected in mice injected with vehicle (Figure 4C).

To confirm that the observed recombination was restricted to astrocytes, we performed EGFP staining in primary astrocytic cultures from pSico-shRNA3 × GFAP-CreERT2 mice. In these cultures, activation of CreERT2 by 4-hydroxytamoxifen, a metabolite that is more efficient than tamoxifen in dissociated cells, induced optimal SR recombination and suppressed EGFP expression as observed by a loss of EGFP signal (Figure 5C). In contrast, no recombination was detected in astrocytes in the absence of 4-hydroxytamoxifen, or in astrocytes from mice carrying only pSico-shRNA3 or GFAP-CreERT2 (Figure 5B).

#### Inducible and constitutive neuron-specific gene silencing in the adult mouse brain

**Figure 6:**
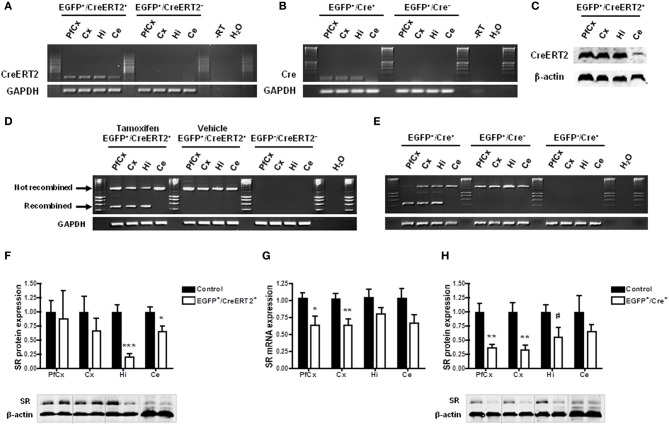
**Constitutive and inducible neuron-specific gene knockdown in the adult mouse brain. (A,B)** RT-PCR showing Cre expression in pSico-shRNA3 × CaMKIIα-CreERT2 (EGFP^+^/CreERT2^+^) **(A)** and pSico-shRNA3 × CaMKIIα-Cre (EGFP^+^/Cre^+^) forebrain structures **(B)**. No expression is detected in the absence of Cre (EGFP^+^/CreERT2^−^ and EGFP^+^/Cre^−^ control mice). –RT, Non-reverse transcribed sample. **(C)** Immunoblot analysis of CreERT2 protein level in pSico-shRNA3 × CaMKIIα-CreERT2 (EGFP^+^/CreERT2^+^) mice. β-actin was used as a loading control. **(D)** Cre-mediated recombination is induced by tamoxifen treatment in different pSico-shRNA3 × CaMKIIα-CreERT2 brain structures. No recombination occurs in vehicle treated pSico-shRNA3 × CaMKIIα-CreERT2 mice. EGFP^−^/CreERT2^−^ mice were used as negative control. **(E)** Cre is constitutively active and leads to recombination in pSico-shRNA3 × CaMKIIα-Cre (EGFP^+^/Cre^+^) forebrain structures, whereas no recombination occurs in mice lacking Cre (EGFP^+^/Cre^−^) or pSico-shRNA3 (EGFP^−^/Cre^+^). GAPDH was used as loading control. **(F)** Immunoblot analysis showing decreased SR level in hippocampus and cerebellum of pSico-shRNA3 × CaMKIIα-CreERT2 adult mice upon tamoxifen treatment (EGFP^+^/CreERT2^+^, *n* = 5). Quantification relative to control mice (EGFP^+^/CreERT2^−^ and EGFP^−^/CreERT2^+^, *n* = 8). 15 μg of total proteins were loaded for prefrontal cortex (PfCx), cortex (Cx), hippocampus (Hi), and 25 μg for cerebellum (Ce). β-actin was used as loading control. **(G)** Real-time quantitative RT-PCR showing significantly decreased SR mRNA expression in prefrontal cortex and cortex of pSico-shRNA3 × CaMKIIα-Cre adult mice (EGFP^+^/Cre^+^, *n* = 8) compared to control mice (EGFP^+^/Cre^−^ and EGFP^−^/Cre^+^, *n* = 11). β-actin was used as an internal control. **(H)** Immunoblot analysis showing decreased SR level in PfCx, Cx, and Hi of pSico-shRNA3 × CaMKIIα-Cre adult mice (EGFP^+^/Cre^+^, *n* = 8). Quantification relative to control mice (EGFP^+^/Cre^−^ and EGFP^−^/Cre^+^, *n* = 8). 15 μg of total proteins were loaded for PfCx, Cx, Hi, and 25 μg for cerebellum (Ce). β-actin was used as loading control. ^#^*p* = 0.07, ^*^p < 0.05, ^**^p < 0.01, ^***^p < 0.001.

We next tested whether the mice carrying pSico-shRNA3 also allow SR knockdown in neurons. For this, we crossed them with transgenic mice expressing CreERT2 (line 1302, unpublished) or Cre (line 2834) (Schweizer et al., 2003) under the control of the forebrain neuron-specific CaMKIIα promoter (both lines generated by conventional pronuclear injection) (Figure 2A and Table S2B). The resulting double mutant animals carry pSico-shRNA3 and either one of Cre transgenes, and are expected to have SR knockdown only in forebrain neurons. Quantitative RT-PCR revealed that in these mice, CreERT2 or Cre was expressed in prefrontal cortex, cerebral cortex, and hippocampus (Figures 6A,B), and led to gene recombination in these areas (Figure 6D), consistent with the forebrain specificity of the CaMKIIα promoter (Mayford et al., 1996; Schweizer et al., 2003). However, CreERT2 was also detected at low level in the cerebellum (Figures 6A,C), as shown by a previous report (Erdmann et al., 2007), but did not induce any recombination. There was also no recombination in mice injected with vehicle, demonstrating tight control of CreERT2 activity by tamoxifen. Recombination led to a significant reduction in the level of SR protein (79.8 ± 28% in hippocampus and 34.8 ± 6% in cerebellum) (Figure 6F). However, SR expression was not altered in the prefrontal and cerebral cortex despite evidence for recombination, suggesting again a dissociation between CreERT2 expression and DNA recombination. SR expression was nonetheless reduced in the olfactory bulb and striatum (Figure S2B). In pSico-shRNA3 × CaMKIIα-Cre mice, DNA recombination was also observed in forebrain structures and correlated with the level and pattern of Cre expression (Figure 6E), as previously observed (Schweizer et al., 2003). This was associated with a significant decrease in SR mRNA level in prefrontal (36.1 ± 7.8%) and cerebral cortex (35.3 ± 5%) when compared to wild-type mice. As expected, SR mRNA was not changed in the cerebellum; unexpectedly, it was not changed in the hippocampus despite obvious DNA recombination in this region (Figure 6G). Consistent with the pattern of mRNA expression, SR protein was significantly reduced in prefrontal cortex (62.3 ± 9%) and cortex (66 ± 16%), but not significantly in cerebellum or hippocampus (Figure 6H). In conclusion, the reduction in SR mRNA and protein obtained in pSico-shRNA3 × CaMKIIα-CreERT2 and pSico-shRNA3 × CaMKIIα-Cre mice suggest a silencing mechanism mediated by shRNA and involving mRNA degradation (McManus et al., 2002; Song et al., 2004).

**Figure 7:**
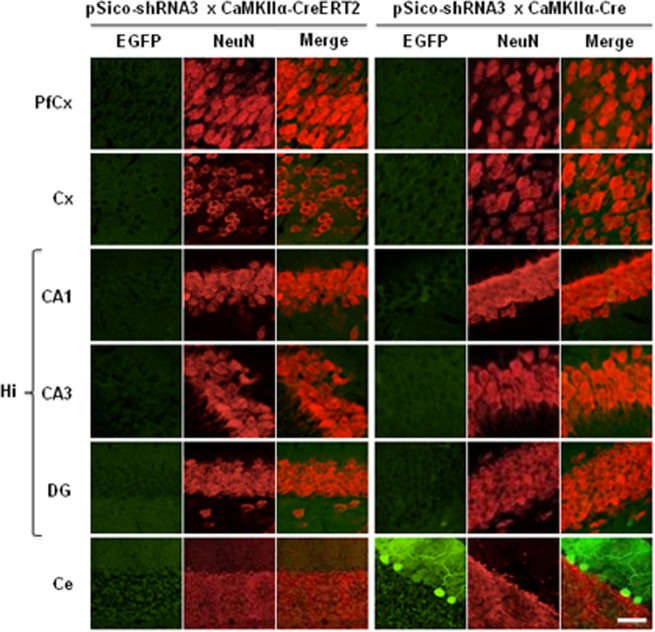
**Visualization of Cre-mediated recombination in neurons.** EGFP (green) and NeuN (red) co-immunostaining in prefrontal cortex (PfCx), cortex (Cx), hippocampus (Hi: CA1, CA3, DG), and cerebellum (Ce). Sections from tamoxifen-treated pSico-shRNA3 × CaMKIIα-CreERT2 and pSico-shRNA3 × CaMKIIα-Cre mice were imaged by Laser Scanning Confocal Microscopy (LSCM). Decreased EGFP expression is observed in neurons from all structures expressing Cre in pSico-shRNA3 × CaMKIIα-CreERT2 and pSico-shRNA3 × CaMKIIα-Cre mice. Scale bar: 10 and 20 μm for cerebellum.

Next, we addressed the cell specificity of DNA recombination and SR knockdown. DNA recombination visualized by a loss of EGFP signal was observed selectively in neurons in prefrontal cortex, cerebellar cortex, and hippocampus (CA1, CA3, and DG) in pSico-shRNA3 × CaMKIIα-CreERT2 treated with tamoxifen and in pSico-shRNA3 × CaMKIIα-Cre mice (Figures 7, S3). However, EGFP fluorescence was not fully eliminated in CA1/CA3 pyramidal neurons and DG granular cells in pSico-shRNA3 × CaMKIIα-Cre mice, suggesting incomplete recombination in these neurons. This may explain why the reduction in SR level in the hippocampus was not significant in this line. A loss of EGFP fluorescence was also detected in Purkinje cells and granule neurons in the cerebellum in pSico-shRNA3 × CaMKIIα-CreERT2 mice (Figures 7, S3). It is also possible that recombination occurred in other neurons expressing EGFP only weakly, but was below detection level.

Overall, these data demonstrate the efficacy of our combined system for inducible or constitutive astrocyte- or neuron-specific gene knockdown in the adult mouse brain.

### Lentiviral transduction of astrocytes and neurons *in vitro*, and in the mouse brain *in vivo*

Lentiviruses have been successfully used in cell culture and in the brain *in vivo* for gene delivery in non-dividing cells (Naldini et al., 1996; Bemelmans et al., 2006; Sapru et al., 2006; Szulc et al., 2006). We examined whether, in addition to being efficient for transgenesis, the pSico-shRNA3 lentivirus was also suitable for cell infection *in vitro*, and in the adult mouse brain *in vivo*. We evaluated pSico-shRNA3 efficacy and cellular tropism using EGFP expression. In cell culture, the virus showed a high infection rate and broad tropism, and led to EGFP expression in primary neurons, mixed primary neuronal cultures and primary astrocytes 24 h after infection (Figures S4A–C). Likewise *in vivo*, the stereotactic injection of pSico-shRNA3 led to EGFP expression 6 weeks after injection in the hippocampus (Figures S5A,B), and 10 days after injection in the striatum (Figure S5C). Similarly, when injected in the rat brain *in vivo*, pSico-shRNA3 showed high cellular transduction in both astrocytes and neurons (data not shown), indicating its efficacy and usefulness in different species.

## Discussion

Here, we report the establishment of a combinatorial genetic approach for heritable gene silencing in the mouse brain *in vivo*. The method is based on the combination of Cre/loxP technology and a lentiviral vector encoding a loxP-controlled shRNA, allowing inducible knockdown of specific target genes in astrocytes or neurons in the adult mouse brain. Because this system uses independent mouse lines, it is versatile and can be applied to any candidate gene in different cell types and brain regions. It can exploit existing shRNA libraries covering mammalian transcripts (Paddison et al., 2004; Root et al., 2006) and any available CreERT2 or Cre transgenic line (Gaveriaux-Ruff and Kieffer, 2007) (see http://jaxmice.jax.org/research/cre/strainlist.html or http://nagy.mshri.on.ca/cre/Search.php for Jackson and Nagy databases). In this study, we generated a novel line expressing CreERT2 in forebrain neurons. This CaMKIIα promoter-CreERT2 line is of particular interest because it allows more extended recombination than a previously reported line (Erdmann et al., 2007).

This study demonstrates the usefulness of a dual system for parallel recombination in astrocytes or neurons in different mice, for comparative analyses of the impact of gene knockdown in these two cell types. Such cellular distinction is instrumental for *in vivo* functional studies of genes whose role in astrocytes or neurons is not known, such as the selected candidate, SR. Together with the inducibility of the knockdown, this cellular specificity provides a major advantage over conventional knock-out methods. It has the potential to limit compensatory effects and circumvent the potential negative effects of gene knockdown on developmental processes, although compensatory effects can potentially still occur after inducible expression (Alvarez et al., 2006). Further, the fact that the candidate gene is knocked-down, rather than knocked-out, is advantageous when trying to recapitulate a disease state caused by hypomorphic mutation(s). It is also an advantage for dissecting out the function of candidate genes whose full deficiency can alter vital cellular processes and possibly induce cell death.

Another advantage of this approach is the rapidity of the generation of the mutant mice resulting from the high efficiency of lentivirus-mediated transgenesis, and its high rate of success compared to conventional pronuclear microinjection (Gordon et al., 1980; Auerbach, 2004). Thus, oocyte survival (92/98, 94% vs. 60–80% for pronuclear injection) and the fraction of injected oocytes resulting in born pups (23/98, 23% vs. 10–20% in classical transgenesis) are overall higher than usually achieved with pronuclear microinjection (Auerbach, 2004). The integration rate of the transgene is also higher [8/11 pseudofounders (72.7%) for pSico-shRNA3 vs. 20–25% (Auerbach, 2004) i.e., 12/55 pseudofounders for CaMKIIα-CreERT2]. Injection into the perivitelline space of fertilized zygotes is also technically less challenging, and is more amenable to application to different mouse strains such as C57BL/6, to genetically engineered oocytes (transgenic GFAP-CreERT2 here), and to other species such as rat. Part of its efficiency is attributed to the rapid and multiple integration rate of lentiviral vectors (Naldini et al., 1996) particularly in dividing cells (Park et al., 2000), which results in the generation of multiple founders in a single injection (Lois et al., 2002). We observed an overall high rate of segregation of the pSico-shRNA3 vector in the eight F1 mouse lines studied, which allowed us to isolate single transgene loci within a few backcrosses (about three). The characteristics of each mouse line regarding transgene integration site(s) and copy number have not been systematically analyzed, thus the influence of the integration site on expression and the stability of the transgene over generations cannot be predicted for each line. Such parameters however need to be determined for gene function studies when using this approach.

Further to demonstrating the usefulness of the pSico construct for lentivirus-mediated transgenesis, this study also establishes its utility in cell culture, and in the brain *in vivo* after stereotactic injection*.* Such different applications open up the possibility to carry out local, cell-specific, and stable gene knockdown in any mice expressing Cre as a transgene, or delivered by other means such as viral vectors (Sinnayah et al., 2004). The efficiency of pSico in primary neuronal and astroglial cultures further demonstrates its cellular tropism in these cell types. Such tropism is postulated to originate from the vesicular stomatitis virus glycoprotein (VSV-G) envelope, found in human immunodeficiency virus type 1 (HIV-1), used for the pSico lentivirus (Li et al., 2012). The U6 and CMV promoters used in the pSico construct show different cellular specificity between brain regions (Makinen et al., 2006), possibly due to variegation effects. It is therefore possible that some EGFP-negative cells express shRNA3 upon Cre-dependent gene recombination. Conversely, some EGFP-positive cells may not express shRNA3 despite Cre-dependent gene recombination. Although Cre-dependent gene recombination with loss of EGFP fluorescence can be partially visualized, Cre-dependent gene recombination and shRNA expression need to be independently verified, as done in this study. Immunostaining using a Cre-specific antibody could be an appropriate alternative to examine the spatial distribution of Cre expression. The dissociation between CreERT2 expression, DNA recombination, and protein knockdown observed in this study may be explained by different factors including variable level of Cre expression, different recombination efficiency, or the presence of Cre-recombination episomal products (Hirrlinger et al., 2006).

While gene knockdown based on siRNA expression from a plasmid encoding shRNA has great potential *in vivo*, many important steps need to be considered for selective and efficient RNAi (Cullen, 2006). First, proper design and selection of specific siRNAs is essential, and has to follow strict rules (Reynolds et al., 2004; Grimm et al., 2006; Pei and Tuschl, 2006). These rules are generally implemented in publicly available softwares for shRNA or siRNA design, but need to be carefully followed. Second, since silencing efficacy *in vivo* is difficult to predict based on *in silico* siRNA design, each siRNA has to be tested and validated *in vitro* before use *in vivo*. Such validation also serves to exclude potential compensatory mechanisms or unspecific responses (Scacheri et al., 2004) (off-target effects) resulting for instance from the activation of interferon pathways (Bridge et al., 2003). Here, using dsRNAs ≤30 bp, that was previously suggested to circumvent unspecific response (Sledz et al., 2003; Reynolds et al., 2006), helped limit unspecificity. Careful control analyses confirmed that the level of unrelated targets such as GAPDH and β-actin was also not altered, suggesting no major off-target effect of shRNA processing *in vivo*. It may result from the moderate shRNA expression, which may have also prevented cell toxicity. Finally, gene knockdown efficiency may vary from individual to individual and thereby introduce phenotypic variability between individuals. In such case, it may be necessary to quantify the level of gene knock-down in each individual and relate it to phenotypic responses in a way to properly evaluate the effects of the manipulation.

In summary, this study is the first to report a genetic system that allows heritable gene knockdown in astrocytes or neurons in the mouse brain *in vivo*. It has all features of the methodologies combined therein—inducibility, tissue- and cell-type specificity, facilitated monitoring, and easy and multiple applications including transgenesis, stereotactic injection, and *in vitro* transduction of primary cells. These features, in combination with the a growing number of CreERT2 and Cre mouse lines available make this approach an extremely useful tool to study neuronal and brain physiology. Additional guidelines on the use of RNAi technology for gene function studies can also be found in the literature (Moffat et al., 2007).

## Methods

### Constructs, design of short hairpin RNAs, and lentivirus production

SR (GenBank accession number AF148321) was amplified from a mouse cDNA brain library (Ambion PCR-ready mouse brain cDNA) by PCR with the following primers (forward: 5'-CGGGATCCGAGGCAGCAGAGAACCATGT-3' and reverse: 5'-CCCAAGCTTAGAGACAATCTTGCCTGAATTT-3') to generate BamHI and HindIII restriction sites. The PCR product was purified, cloned into pCR2.1-TOPO plasmid (Invitrogen), digested with BamHI and HindIII restriction enzymes, and ligated into pCMV-Tag2 mammalian expression plasmid (Stratagene) to obtain pCMV-Tag2-SR carrying SR cDNA fused to a synthetic epitope peptide (FLAG tag) in N-terminal. pSicoOligomaker 1.5 program (http://web.mit.edu/jacks-lab/protocols/pSico.html) was used to design mouse SR shRNA oligos (see Table S1B). A scrambled shRNA not complementary to any mouse or human sequence was used as control (Flygare et al., 2005). shRNAs 3, 4 and scrambled shRNAs were cloned into pSicoR, and shRNA 3 into pSico (Jacks lab, MIT) according to Jackson's lab protocol to generate pSicoR-shRNA3, pSicoR-shRNA4, pSicoR-shRNAsc, and pSico-shRNA3. Constructs were verified by sequencing. pSico-shRNA3, the packaging encoding vector psPAX2 and the envelope encoding vector pMD2.G (obtained from Dr. Didier Trono, EPFL Lausanne) were used for lentivirus production (see http://tronolab.epfl.ch/page58122.html for details). Viral particles were titrated by FACS analysis of infected Hek293T cells.

### Cell culture and transfection assays

Hek293T cells were cultured in standard conditions and transfected with pCMV-Tag2-SR (50, 100, and 150 ng/~2 × 10^5^ cells), pCMV-Tag2 control (carrying the firefly luciferase gene), pCMV-Tag2 (empty vector; mock plasmid), pSicoR-shRNA3, pSicoR-shRNA4, and pSicoR-shRNAsc plasmids (150 ng/~2 × 10^5^ cells) using FuGENE 6 (Roche; see Table S1B) according to the manufacturer's protocol. siRNA duplexes (Qiagen) were co-transfected (50 and 150 ng/~2 × 10^5^ cells) with pCMV-Tag2-SR (50 ng/~2 × 10^5^ cells) using X-tremeGENE reagent (Roche). Plasmid and siRNA transfection efficiency was evaluated by fluorescence obtained from pSicoR-shRNAs and siRNA Alexa Fluor 488 (Qiagen; 500 ng/~2 × 10^5^ cells), respectively. Primary astroglial cultures were obtained by isolating astrocytes from P6 mouse brains plated on poly-L-lysine-coated (Sigma) glass coverslips and cultivated in DMEM (Gibco) containing 10% fetal bovine serum (Invitrogen).

### Generation of transgenic mice

#### Lentivirus-mediated transgenesis

Transgenic mice carrying pSico-shRNA3 were generated by microinjection of lentiviral particles (~100 pl, 2.5 × 10^8^ TU/ml) into the perivitelline space of fertilized one-cell oocytes isolated from four heterozygous GFAP-CreERT2 superovulated females (98 eggs, FVB/N background provided by Dr. Frank Kirchhoff, Göttingen) (Hirrlinger et al., 2006). At the blastocyst stage (after 3 days in culture *in vitro*), the 92 embryos that survived were implanted into the oviducts of 4 pseudo-pregnant C57BL/6 foster females. Resulting offspring was genotyped by PCR using primers complementary to EGFP (present on pSico-shRNA3). Positive animals were backcrossed to C56BL/6/J mice, or to CaMKIIα-CreERT2 (line 1302, see below) or CaMKIIα-Cre (line 2834) (Schweizer et al., 2003) mice. Genotyping was performed using EGFP F2 (5'-CTA TAT CAT GGC CGA CAA GC-3') and R2 (5'-ACT GGG TGC TCA GGT AGT GG-3') primers for pSico-shRNA3 transgene, and pNN3050 (5'-CGA TTC TAG AAT TCG CTG TCT GC-3') and Cre antisense (5'-CAG GGT GTT ATA AGC AAT CCC-3') primers for CreERT2 and Cre transgene amplification.

#### Classical transgenesis

CaMKIIα-CreERT2 mice were generated by microinjection into fertilized mouse oocytes (C57BL/6/JXDBA hybrid) with a recombinant transgene cloned as follows. CreERT2 cDNA was excised from the vector pBSII SK+ (generously provided by Dr. Philipp Berger) by SpeI/KpnI digestion and inserted into the BamHI site of pNN265 (Michalon et al., 2005). An hFGF2 IRES sequence excised from pCRFL plasmid (gift from Prof. A. C. Prats) by NarI/SpeI digestion was blunt-ended using T4 polymerase (New England Biolabs) and introduced into the SalI site of pNN265 CreERT2. A NotI fragment was excised (5022 bp) from this recombinant vector and introduced into the NotI site of pMM403 containing the CaMKIIα promoter. SfiI digest resulted in a 13,049 bp fragment used for microinjection. 12 founders (out of 55 pseudo-founders) were obtained, 7 of which transmitted the transgene to their offspring, and gave rise to 7 independent lines. F1 animals were backcrossed to C57Bl/6J mice. Line 1302 was used in this study.

### Tamoxifen treatment

Tamoxifen solution was prepared and administered daily as previously described (Hirrlinger et al., 2006). Briefly, tamoxifen (Sigma-Aldrich) was dissolved in 90% corn oil (Sigma-Aldrich) and 10% ethanol to a final concentration of 10 mg/ml. Adult mice received intraperitoneal injections (1 mg) twice a day for 5 days. In primary astrocyte cultures, gene recombination was induced by application of 1 μM 4-hydroxytamoxifen (tamoxifen metabolite) for 48 h. Corn oil/ethanol was used as carrier.

### Western blot analysis

Hek293T cells were harvested 24 and 48 h after transfection and lysed with NP-40 buffer. After centrifugation, soluble and membrane protein fractions were resolved on 10% SDS-PAGE and transferred onto a PVDF membrane (BioRad). Brains were dissected and homogenized using a 26G syringe in 10 mM HEPES, 1 mM MgCl_2_, 5 mM EDTA, 0.2% (v/v) Triton X-100 (Sigma), 10% (v/v) glycerol (Sigma), protease inhibitor cocktail (Sigma), 250 μM PMSF (Sigma), and 15 mM β-mercaptoethanol (Sigma). 15 to 25 μ g total protein was resolved on 10% SDS-PAGE and transferred onto a nitrocellulose membrane (BioRad). Membranes were blocked (Rockland IR blocking buffer, Rockland), and incubated in primary antibodies: anti-mouse flag M2 primary monoclonal antibody (1:5000; Sigma), anti-mouse SR primary antibody (1:500; BD Biosciences), rabbit anti-mouse estrogen receptor α (1:500; Santa Cruz Biotechnology), mouse anti-GFP (1:1000; Invitrogen), mouse anti-mouse β-actin (1:5000; Sigma), or rabbit anti-GAPDH (1:1000; Abcam). Membranes were then incubated in goat anti-mouse IRDye 680 secondary antibody (1:5000; Li-Cor Biosciences). Band intensity was determined and quantified using an Odyssey IR scanner (Li-Cor Biosciences). The protein signal was normalized to β-actin.

### Quantitative real-time RT-PCR

Total RNA from prefrontal cortex, cortex, hippocampus, cerebellum, and primary astrocytes was extracted using a NucleoSpin Kit II (Macherey-Nagel), purified with RQ1 DNase (Promega) and reverse-transcribed using a SuperScript First-Strand Synthesis System for RT-PCR II (Invitrogen). Quantitative PCR was performed with a mouse SR-specific Taqman probe (Mm00489125_m1, Applied Biosystems) and an Applied Biosystems 7500 Thermal Cycler. Each sample was analyzed in triplicate and equal amount of cDNA was plated. Values were chosen in the linear range of amplification, and the comparative Ct method was used to determine differences in gene expression between samples. β-actin was used as an internal control for normalization.

### PCR and RT-PCR

PCR was performed on cDNA (2 μ l) using the primers Cre up (5'-AGG CTA AGT GCC TTC TCT ACA C-3') and Cre lo (5'-ACC AGG TTC GTT CAC TCA TGG-3') for Cre and CreERT2 amplification, and primers EGFP F2 (5'-CTA TAT CAT GGC CGA CAA GC-3') and R2 (5'-ACT GGG TGC TCA GGT AGT GG-3') for EGFP. Gene recombination was assessed by PCR on genomic DNA by using the primers loop-out F (5'-CCC GGT TAA TTT GCA TAT AAT ATT TC-3') and loop-out R (5'-CAT GAT ACA AAG GCA TTA AAG CAG-3') (Ventura et al., 2004). PCR products were resolved on a 2% agarose gel. cDNA and gDNA quality and loading were verified with the housekeeping gene GAPDH using the primers GAPDH F1 (5'-CAC TGA GCA TCT CCC TCA CA-3') and GAPDH R1 (5'-GTG GGT GCA GCG AAC TTT AT-3').

### Immunofluorescence

Animals were sacrificed and transcardially perfused with Ringer solution followed by 4% paraformaldehyde (Sigma) and 15% of a saturated solution of picric acid in phosphate buffer (0.1 M, pH7.4; flow: 20 ml/min). Brains were quickly removed, postfixed in the same solution and transferred into a 30% sucrose solution. Sagital or coronal sections (50 μm thick) were cut with a cryostat. Free-floating brain sections were washed in 0.1 M PB, blocked and permeabilized in 0.1 M PB, 0.4% Triton X-100 (Sigma), and 10% heat-inactivated horse serum (HS; Sigma) for 12 h at 4°C. When needed, slices were heated in 0.1 M Tris pH 8.0, Glycin 50 mM at 80°C for 15 min to unmask the reactivity of EGFP antigens. Slices were then incubated with primary rabbit anti-EGFP (Synaptic Systems), anti-NeuN (Chemicon), and anti-GFAP (Dako) antibodies (1:1000) for 12 h at 4°C in 0.1 M PB, 0.4% Triton X-100, and 10% HS. Slices were washed in 0.1 M PB, 0.4% Triton X-100 and incubated overnight at 4°C with goat anti-rabbit FITC and donkey anti-mouse TRITC fluorescence-conjugated secondary antibodies (1:1000; Jackson ImmunoResearch). After washing in 0.1 M PB, slices were mounted using Mowiol (Molecular Probes) and stored in the dark at 4°C. Primary astroglial cultures were fixed overnight into a 4% paraformaldehyde/phosphate buffer solution (0.1 M, pH 7.4), washed in 0.1 M PB, blocked and permeabilized in 0.1 M PB, 0.4% Triton X-100 (Sigma), and 10% heat-inactivated horse serum (HS; Sigma) for 12 h at 4°C. Low magnification fluorescence images were acquired with a CoolSNAPK4 digital camera (Roper Scientific) mounted on an Axiophot microscope (Zeiss) and analyzed using MCID Elite 7.0 software (MCID). High magnification images were taken with a Zeiss LSM 410 confocal laser-scanning microscope using lasers pretuned to 543 nm (TRITC) and 488 nm (FITC), and images were averaged to improve signal-to-noise ratio.

### Statistical analysis

Data are presented as mean normalized to baseline or control ± SEM. Paired Student's *t*-tests were used to compare non-normalized data. Statistical significance was set at *p* ≤ 0.05(^*^), *p* ≤ 0.01(^**^), and *p* ≤ 0.001(^***^).

## Author contributions

Fabrice Heitz was responsible for the project, prepared figures, and wrote the manuscript. He performed the biochemical and molecular experiments shown in Figures 3–7, and Figures S2, S3, and stereotactic injections used to generate Figure S5 data. Torbjörn Johansson generated all constructs and produced the lentiviruses. He performed experiments shown in Figures 1, S1, S4. Karsten Baumgärtel generated the CaMKIIα-CreERT2 construct used for transgenesis. Rreze Gecaj performed experiments and contributed to the results presented in Figures 4, 6. Pawel Pelczar performed lentivirus and DNA microinjection for transgenesis. Isabelle M. Mansuy conceived the project, provided continual conceptual input and financial support, and participated to the writing process.

## Conflict of interest statement

The authors declare that the research was conducted in the absence of any commercial or financial relationships that could be construed as a potential conflict of interest.

## Supplementary material

Figure S1 **(A)** siRNA3 induces SR knockdown. Hek293T cells were co-transfected with pCMV-Tag2-SR (50 ng) and siRNA3 (50 and 150 ng), siRNA4 (50 ng), or control siRNA (siRNA Alexa Fluor 488, 50 ng). SR level in cells transfected with pCMV-Tag2-SR (50 ng) was used as control. **(B)** Quantification of SR knockdown induced by shRNA3 in three independent experiments (#1, 2, and 3). Hek293T cells were co-transfected with pCMV-Tag2-SR (50 ng) and the empty vector pCDNA3.1 (Control), pSicoR-shRNAsc (50 ng or 150 ng; ratio 1:1 or 3:1), or pSicoR-shRNA3 (50 ng or 150 ng; ratio 1:1 or 3:1). Values are mean % of SR knockdown relative to control normalized to β-actin.

Figure S2 **(A)** EGFP expression in olfactory bulb granular cells (OB) and striatal medium spiny neurons (Str) in pSico-shRNA3 × CaMKIIα-CreERT2 mice, as seen by EGFP and NeuN co-localization. Scale bar: 80 μm (20×) and 20 μm (80×). **(B)** SR expression in olfactory bulb and striatum in pSico-shRNA3 × CaMKIIα-CreERT2 mice (EGFP^+^/CreERT2^+^, *n* = 4). mRNA level measured by qRT-PCR relative to controls (EGFP^+^/CreERT2^−^ and EGFP^−^/CreERT2^+^, *n* = 6).

Figure S3 **Cre-mediated recombination in neurons.** Immunohistochemical analysis of EGFP expression in control mice (pSico-shRNA3) and in tamoxifen-treated pSico-shRNA3 × CaMKIIα-CreERT2 and pSico- shRNA3 × CaMKIIα-Cre adult mice. EGFP (green) and NeuN (red) co-immunostaining in prefrontal cortex (PfCx), cortex (Cx), hippocampus (CA1, CA3, DG), and cerebellum (Ce). Sections were imaged by LSCM. Decreased EGFP expression is observed in neurons in all structures expressing CreERT2 and Cre. Scale bar: 40 μm.

Figure S4 **(A)** Distribution of EGFP (green) 24 h after infection of mouse primary neurons (nuclei stained with DAPI; blue fluorescence) with pSico-shRNA3 lentivirus (2.5 × 10^6^ TU/ml; 3 μl/ml culture medium). Scale bar: 50 μm. **(B)** Lentiviral infection of rat mixed primary neuronal cultures. Cells (14 DIV) were infected with pSico lentivirus (2.5 × 10^8^ TU/ml) constitutively expressing EGFP. One week after infection, neurons were immunolabeled with the neuron-specific marker mitochondrial associated protein-2 (MAP-2), and analyzed for EGFP expression under a confocal microscope. Arrows indicate neurons (MAP-2/red) stably expressing EGFP (red and green overlay). Scale bar: 20 μm. **(C)** Lentiviral infection of primary astrocytes. Astrocytes were infected with pSicoR-shRNA3 lentivirus (1.4 × 10^8^ TU/ml; 20 μl/ml culture medium). Seven days after infection, cells were immunolabeled for GFAP, EGFP, and the nuclear marker DAPI (blue dots in merge), and visualized under a fluorescence microscope. Scale bar: 160 μm

Figure S5 **(A)** Coronal section of mouse ventral hippocampus (posterior) stereotactically injected with pSicoR-shRNA3 lentivirus. Brains were isolated 6 weeks after injection, and fresh-frozen sections were collected then analyzed under a fluorescence microscope. A: Ipsilateral hemisphere injected with 2 μl lentivirus pSicoR-shRNA3 (6 × 10^8^ TU/ml); Ventral part coordinates (relative to bregma): AP −3.16 mm, ML ±3.00 mm, DV −3 to −2 mm; dorsal part coordinates: AP −2.30 mm, ML ±2.00, DV −1.5 to −1.25 mm; volume flow: 0.5 μ l.min^−1^). B: Contralateral non-injected hemisphere (enhanced contrast for identification of hippocampal structures). Scale bar: 160 μm. **(B)** Western blot analysis of EGFP expression in hippocampal homogenates three weeks post-injection. β-actin (bottom) was used as loading control. Control, non-injected hemisphere. **(C)** Regional distribution of transduced cells in the medial striatum 10 days following stereotactic injection of 2 μl pSicoR-shRNA3 lentivirus (6 × 10^8^ TU/ml; coordinates (relative to bregma): AP 0.50 mm, ML ±2.00 mm, DV: −3 to −2 mm; volume flow: 0.5 μ l.min^−1^), as revealed by EGFP fluorescence. Left inset: localization of the injection site. Scale bar: 500 μm.

Table S1 **Double-stranded siRNAs and oligonucleotides for SR silencing. (A)** Four different siRNAs (1–4) complementary to SR mRNA sequence (target) were designed. Sense and antisense siRNAs are represented. siRNA Alexa Fluor 488 negative control: siRNA with no homology to any known mammalian gene and labeled with Alexa Fluor 488 fluorescent dye for transfection monitoring and optimization. **(B)** siRNA3 and siRNA4-based oligos 3 and 4 with targeted SR cDNA sequence. Sense and antisense sequences for shRNA expression with loop sequence TTCAAGAGA and 5' terminal Ts (on sense strand). Predicted sense and antisense siRNAs after shRNA processing are in bold. Scrambled oligo coding for an shRNA not targeting any mammalian gene was used as negative control.

Table S2 **Germline transmission of the proviral transgene pSico-shRNA3 in pSico-shRNA3 × GFAP-CreERT2, CaMKIIα-CreERT2, and CaMKIIα-Cre mice.** The yield of PCR-positive and PCR-negative mice is presented as a ratio with the corresponding percentage of pSico-shRNA3 and CreERT2 or Cre transgene segregation. **(A)** Eight mouse lines were obtained from pSico-shRNA3 × GFAP-CreERT2 F0 founders (5 females and 2 males) and backcrossed down to 5 generations. **(B)** Transgene segregation in pSico-shRNA3 × CaMKIIα-CreERT2 (F1–5) and pSico-shRNA3 × CaMKIIα-Cre mice (F1–3). F1 pSico-shRNA3 × CaMKIIα-CreERT2 mice (line 1 and 2) were generated by breeding pSico-shRNA^+^/GFAP-CreERT2^−^ F2 mice to CaMKIIα-CreERT2 mice (line 1302 females). F1 pSico- shRNA3 × CaMKIIα-CreERT2 mice (line 1) were generated from pSico-shRNA^+^/GFAP-CreERT2^−^ F4 mice (two line 2 females) bred to a CaMKIIα-Cre mouse (line 2834 male). The pSico-shRNA3 construct was reliably transmitted in F1–F5 progeny, indicating stable integration and effective transmission across generations.
