# Supplementary material for: Heritable and inducible gene knockdown in astrocytes or neurons in vivo by a combined lentiviral and RNAi approach
Source: Front Cell Neurosci. 2014 Mar 19;8:62. doi: 10.3389/fncel.2014.00062 (PMC3958736; doi:10.3389/fncel.2014.00062)
Supplement: Figure S1 — (A) siRNA3 induces SR knockdown. Hek293T cells were co-transfected with pCMV-Tag2-SR (50 ng) and siRNA3 (50 and 150 ng), siRNA4 (50 ng), or control siRNA (siRNA Alexa Fluor 488, 50 ng). SR level in cells transfected with pCMV-Tag2-SR (50 ng) was used as control. (B) Quantification of SR knockdown induced by shRNA3 in three independent experiments (#1, 2, and 3). Hek293T cells were co-transfected with pCMV-Tag2-SR (50 ng) and the empty vector pCDNA3.1 (Control), pSicoR-shRNAsc (50 ng or 150 ng; ratio 1:1 or 3:1), or pSicoR-shRNA3 (50 ng or 150 ng; ratio 1:1 or 3:1). Values are mean % of SR knockdown relative to control normalized to β-actin. [file Presentation1.ZIP › Supplementary Methods.pdf]

Supplementary Methods

Primary cell cultures

Mouse hippocampal neurons were cultured as previously described (Buerli et al., 2007). Infection with pSico-shRNA3 (2.5 x 10^6 TU/ml; 3 µl/ml culture medium) was performed at DIV11. Neurons were fixed 24 h post-infection in 4% PFA and stained with EGFP and DAPI (see Methods section). Rat primary mixed neuronal cultures were prepared as described previously (Brunig et al., 2002) and infected with pSico (2.5 x 10^8 TU/ml) at DIV14. Fixation and immunostaining were done 1 week later. Images were taken using a Zeiss LSM 410 confocal laser-scanning microscope.

Stereotactic injection

Adult mice and rats were anaesthetized by isoflurane inhalation (5% isoflurane and 95% oxygen) and sedation was maintained through the whole procedure. A micro-hole was drilled in the skull and a catheter connected to an electronic pump gently placed. Lentiviral preparation was slowly infused (up to 2 µl/injection).

Supplementary References

I. Brunig, A. Suter, I. Knuesel, B. Luscher and J. M. Fritschy: GABAergic terminals are required for postsynaptic clustering of dystrophin but not of GABA(A) receptors and gephyrin. *J Neurosci*, 22(12), 4805-13 (2002) doi:22/12/4805
T. Buerli, C. Pellegrino, K. Baer, B. Lardi-Studler, I. Chudotvorova, J. M. Fritschy, I. Medina and C. Fuhrer: Efficient transfection of DNA or shRNA vectors into neurons using magnetofection. *Nat Protoc*, 2(12), 3090-101 (2007)
