# Supplementary figures and images for: Heritable and inducible gene knockdown in astrocytes or neurons in vivo by a combined lentiviral and RNAi approach
Source: Front Cell Neurosci. 2014 Mar 19;8:62. doi: 10.3389/fncel.2014.00062 (PMC3958736; doi:10.3389/fncel.2014.00062)

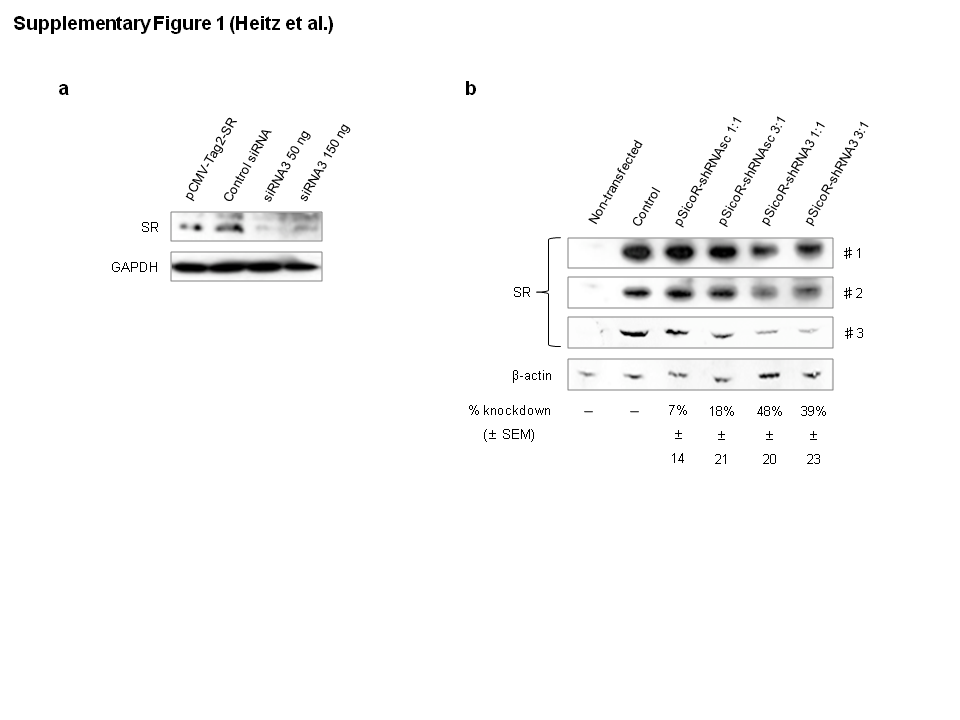

Supplement: Figure S1 — (A) siRNA3 induces SR knockdown. Hek293T cells were co-transfected with pCMV-Tag2-SR (50 ng) and siRNA3 (50 and 150 ng), siRNA4 (50 ng), or control siRNA (siRNA Alexa Fluor 488, 50 ng). SR level in cells transfected with pCMV-Tag2-SR (50 ng) was used as control. (B) Quantification of SR knockdown induced by shRNA3 in three independent experiments (#1, 2, and 3). Hek293T cells were co-transfected with pCMV-Tag2-SR (50 ng) and the empty vector pCDNA3.1 (Control), pSicoR-shRNAsc (50 ng or 150 ng; ratio 1:1 or 3:1), or pSicoR-shRNA3 (50 ng or 150 ng; ratio 1:1 or 3:1). Values are mean % of SR knockdown relative to control normalized to β-actin. [file Presentation1.ZIP › 78314_Mansuy_Suppl_Figure_1.TIF]

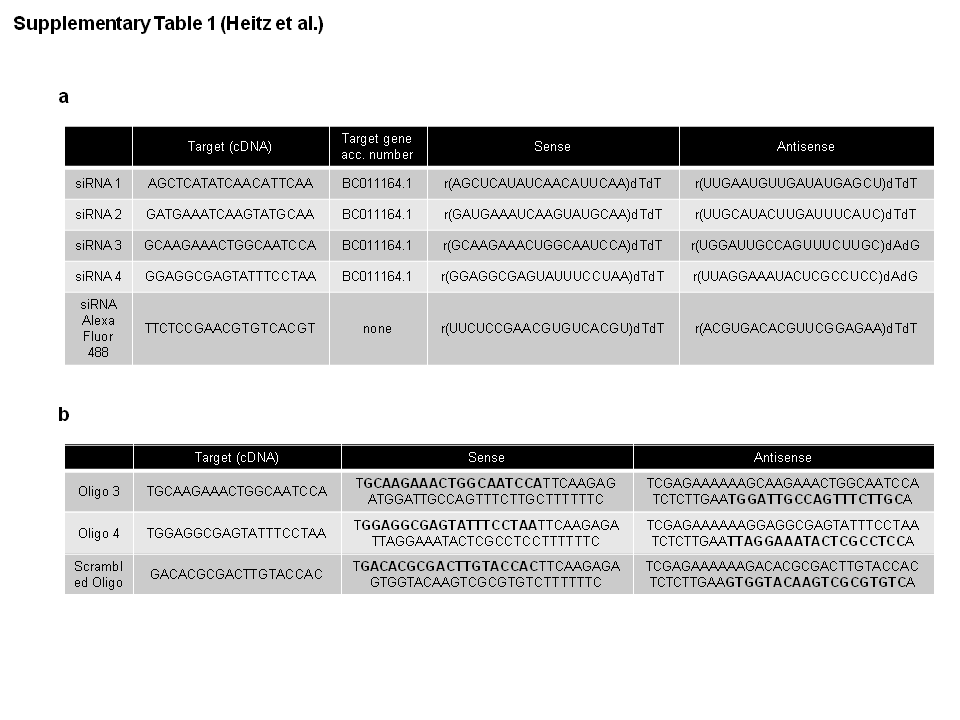

Supplement: Figure S1 — (A) siRNA3 induces SR knockdown. Hek293T cells were co-transfected with pCMV-Tag2-SR (50 ng) and siRNA3 (50 and 150 ng), siRNA4 (50 ng), or control siRNA (siRNA Alexa Fluor 488, 50 ng). SR level in cells transfected with pCMV-Tag2-SR (50 ng) was used as control. (B) Quantification of SR knockdown induced by shRNA3 in three independent experiments (#1, 2, and 3). Hek293T cells were co-transfected with pCMV-Tag2-SR (50 ng) and the empty vector pCDNA3.1 (Control), pSicoR-shRNAsc (50 ng or 150 ng; ratio 1:1 or 3:1), or pSicoR-shRNA3 (50 ng or 150 ng; ratio 1:1 or 3:1). Values are mean % of SR knockdown relative to control normalized to β-actin. [file Presentation1.ZIP › 78314_Mansuy_Suppl_Table 1.TIF]

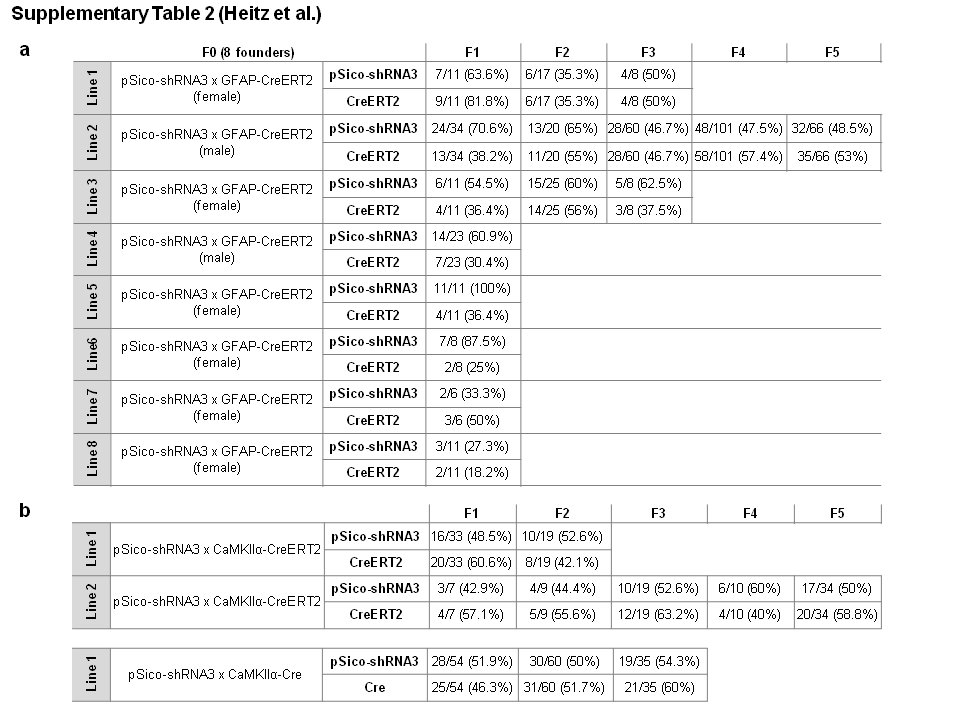

Supplement: Figure S1 — (A) siRNA3 induces SR knockdown. Hek293T cells were co-transfected with pCMV-Tag2-SR (50 ng) and siRNA3 (50 and 150 ng), siRNA4 (50 ng), or control siRNA (siRNA Alexa Fluor 488, 50 ng). SR level in cells transfected with pCMV-Tag2-SR (50 ng) was used as control. (B) Quantification of SR knockdown induced by shRNA3 in three independent experiments (#1, 2, and 3). Hek293T cells were co-transfected with pCMV-Tag2-SR (50 ng) and the empty vector pCDNA3.1 (Control), pSicoR-shRNAsc (50 ng or 150 ng; ratio 1:1 or 3:1), or pSicoR-shRNA3 (50 ng or 150 ng; ratio 1:1 or 3:1). Values are mean % of SR knockdown relative to control normalized to β-actin. [file Presentation1.ZIP › 78314_Mansuy_Suppl_Table 2.TIF]

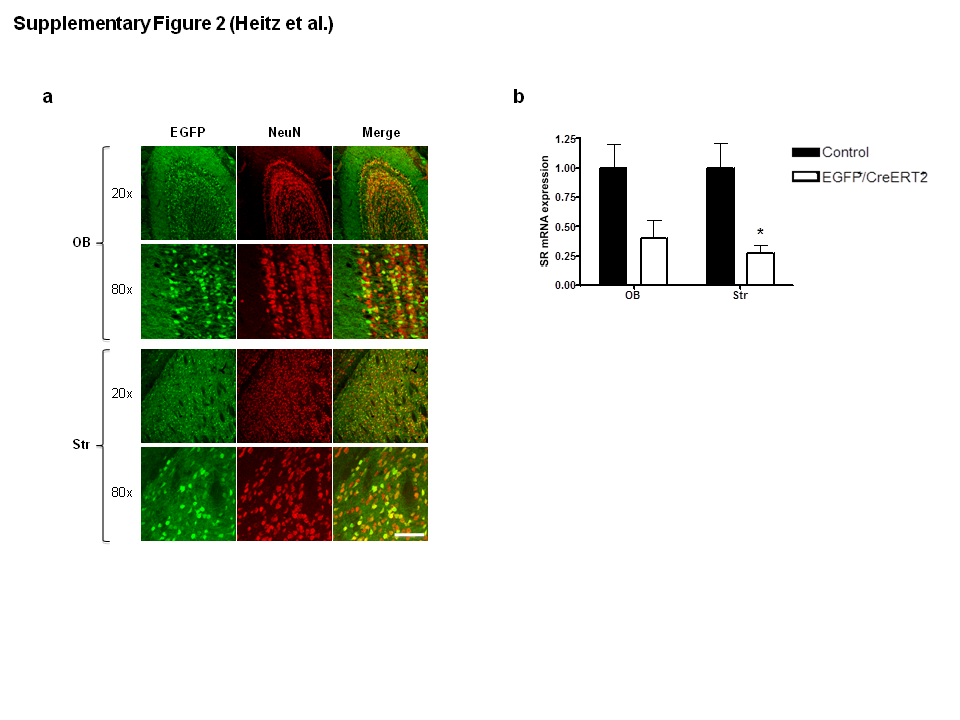

Supplement: Figure S1 — (A) siRNA3 induces SR knockdown. Hek293T cells were co-transfected with pCMV-Tag2-SR (50 ng) and siRNA3 (50 and 150 ng), siRNA4 (50 ng), or control siRNA (siRNA Alexa Fluor 488, 50 ng). SR level in cells transfected with pCMV-Tag2-SR (50 ng) was used as control. (B) Quantification of SR knockdown induced by shRNA3 in three independent experiments (#1, 2, and 3). Hek293T cells were co-transfected with pCMV-Tag2-SR (50 ng) and the empty vector pCDNA3.1 (Control), pSicoR-shRNAsc (50 ng or 150 ng; ratio 1:1 or 3:1), or pSicoR-shRNA3 (50 ng or 150 ng; ratio 1:1 or 3:1). Values are mean % of SR knockdown relative to control normalized to β-actin. [file Presentation1.ZIP › 78314_Mansuy_Suppl_Figure 2.TIF]

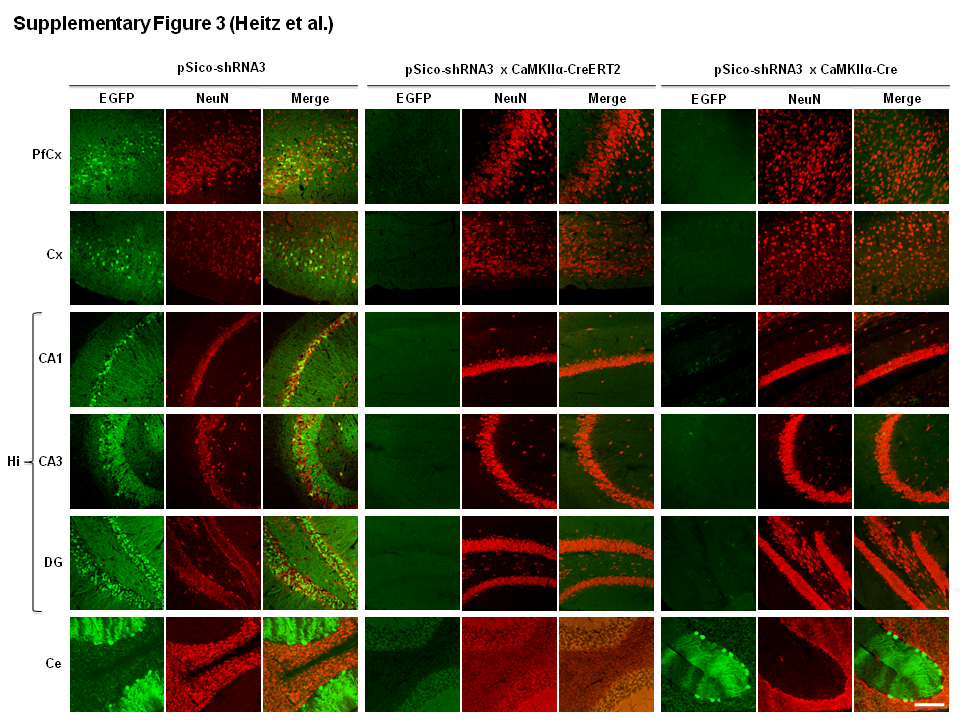

Supplement: Figure S1 — (A) siRNA3 induces SR knockdown. Hek293T cells were co-transfected with pCMV-Tag2-SR (50 ng) and siRNA3 (50 and 150 ng), siRNA4 (50 ng), or control siRNA (siRNA Alexa Fluor 488, 50 ng). SR level in cells transfected with pCMV-Tag2-SR (50 ng) was used as control. (B) Quantification of SR knockdown induced by shRNA3 in three independent experiments (#1, 2, and 3). Hek293T cells were co-transfected with pCMV-Tag2-SR (50 ng) and the empty vector pCDNA3.1 (Control), pSicoR-shRNAsc (50 ng or 150 ng; ratio 1:1 or 3:1), or pSicoR-shRNA3 (50 ng or 150 ng; ratio 1:1 or 3:1). Values are mean % of SR knockdown relative to control normalized to β-actin. [file Presentation1.ZIP › 78314_Mansuy_Suppl_Figure 3.TIF]

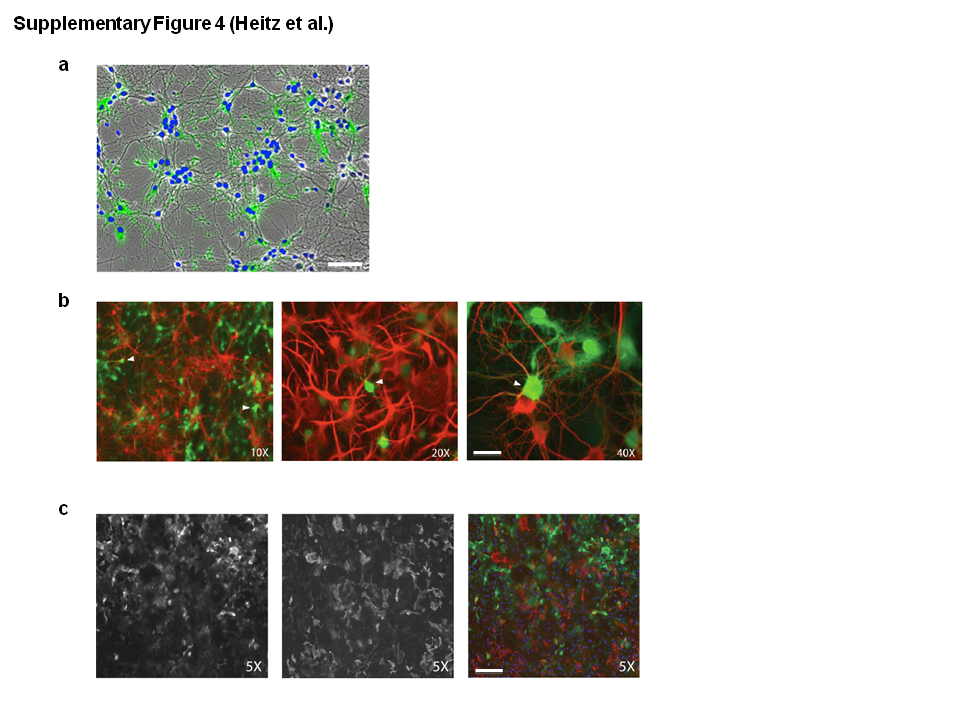

Supplement: Figure S1 — (A) siRNA3 induces SR knockdown. Hek293T cells were co-transfected with pCMV-Tag2-SR (50 ng) and siRNA3 (50 and 150 ng), siRNA4 (50 ng), or control siRNA (siRNA Alexa Fluor 488, 50 ng). SR level in cells transfected with pCMV-Tag2-SR (50 ng) was used as control. (B) Quantification of SR knockdown induced by shRNA3 in three independent experiments (#1, 2, and 3). Hek293T cells were co-transfected with pCMV-Tag2-SR (50 ng) and the empty vector pCDNA3.1 (Control), pSicoR-shRNAsc (50 ng or 150 ng; ratio 1:1 or 3:1), or pSicoR-shRNA3 (50 ng or 150 ng; ratio 1:1 or 3:1). Values are mean % of SR knockdown relative to control normalized to β-actin. [file Presentation1.ZIP › 78314_Mansuy_Suppl_Figure 4.TIF]

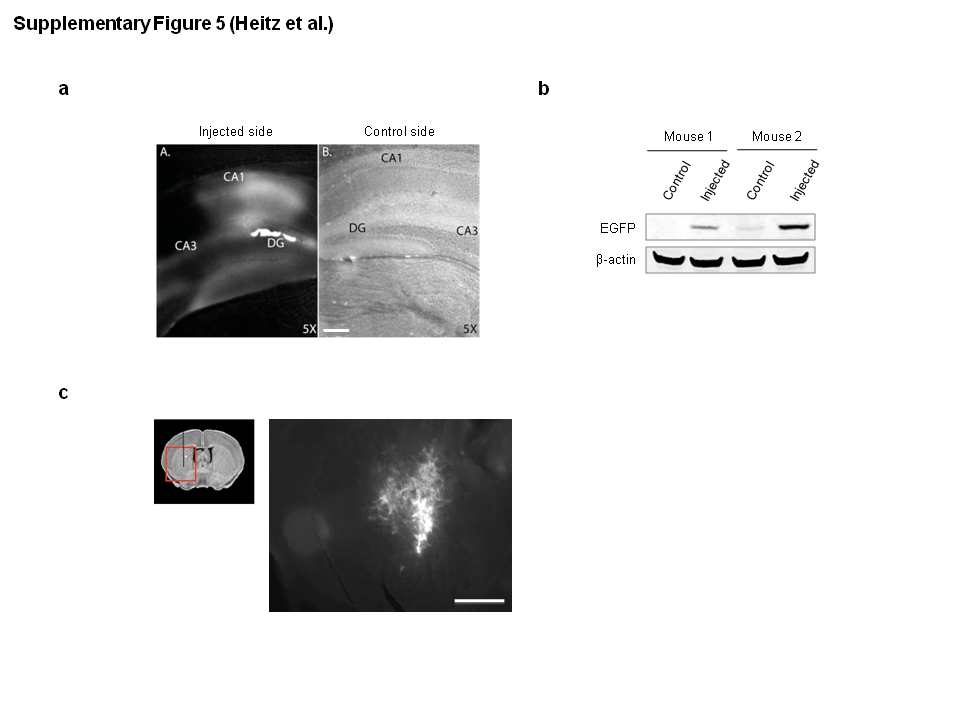

Supplement: Figure S1 — (A) siRNA3 induces SR knockdown. Hek293T cells were co-transfected with pCMV-Tag2-SR (50 ng) and siRNA3 (50 and 150 ng), siRNA4 (50 ng), or control siRNA (siRNA Alexa Fluor 488, 50 ng). SR level in cells transfected with pCMV-Tag2-SR (50 ng) was used as control. (B) Quantification of SR knockdown induced by shRNA3 in three independent experiments (#1, 2, and 3). Hek293T cells were co-transfected with pCMV-Tag2-SR (50 ng) and the empty vector pCDNA3.1 (Control), pSicoR-shRNAsc (50 ng or 150 ng; ratio 1:1 or 3:1), or pSicoR-shRNA3 (50 ng or 150 ng; ratio 1:1 or 3:1). Values are mean % of SR knockdown relative to control normalized to β-actin. [file Presentation1.ZIP › 78314_Mansuy_Suppl_Figure 5.TIF]
